# Trajectories of depressive symptoms and adult educational and employment outcomes

**DOI:** 10.1192/bjo.2019.90

**Published:** 2019-12-12

**Authors:** José A. López-López, Alex S. F. Kwong, Elizabeth Washbrook, Rebecca M. Pearson, Kate Tilling, Mina S. Fazel, Judi Kidger, Gemma Hammerton

**Affiliations:** Assistant Professor, Department of Basic Psychology and Methodology, University of Murcia, Spain; Honorary Research Fellow, Department of Population Health Sciences, Bristol Medical School; and Centre for Academic Mental Health, University of Bristol, UK; Student, School of Geographical Sciences, Centre for Multilevel Modelling and MRC Integrative Epidemiology Unit, University of Bristol, UK; Associate Professor in Quantitative Methods, Centre for Multilevel Modelling and School of Education, University of Bristol, UK; Lecturer in Psychiatric Epidemiology, Department of Population Health Sciences, Bristol Medical School; and Centre for Academic Mental Health, University of Bristol, UK; Professor of Medical Statistics, Department of Population Health Sciences, Bristol Medical School; and MRC Integrative Epidemiology Unit, University of Bristol, UK; Associate Professor, Department of Psychiatry, University of Oxford, UK; Lecturer in Public Health, Department of Population Health Sciences, Bristol Medical School; and Centre for Academic Mental Health, University of Bristol, UK; Senior Research Associate, Department of Population Health Sciences, Bristol Medical School; and Centre for Academic Mental Health, University of Bristol, UK

**Keywords:** Depressive disorders, education and training, epidemiology, statistical methodology

## Abstract

**Background:**

Depressive symptoms show different trajectories throughout childhood and adolescence that may have different consequences for adult outcomes.

**Aims:**

To examine trajectories of childhood depressive symptoms and their association with education and employment outcomes in early adulthood.

**Method:**

We estimated latent trajectory classes from participants with repeated measures of self-reported depressive symptoms between 11 and 24 years of age and examined their association with two distal outcomes: university degree and those not in employment, education or training at age 24.

**Results:**

Our main analyses (*n* = 9399) yielded five heterogenous trajectories of depressive symptoms. The largest group found (70.5% of participants) had a stable trajectory of low depressive symptoms (stable–low). The other four groups had symptom profiles that reached full-threshold levels at different developmental stages and for different durations. We identified the following groups: childhood–limited (5.1% of participants) with full-threshold symptoms at ages 11–13; childhood–persistent (3.5%) with full-threshold symptoms at ages 13–24; adolescent onset (9.4%) with full-threshold symptoms at ages 17–19; and early-adult onset (11.6%) with full-threshold symptoms at ages 22–24. Relative to the majority ‘stable–low’ group, the other four groups all exhibited higher risks of one or both adult outcomes.

**Conclusions:**

Accurate identification of depressive symptom trajectories requires data spanning the period from early adolescence to early adulthood. Consideration of changes in, as well as levels of, depressive symptoms could improve the targeting of preventative interventions in early-to-mid adolescence.

## Background

Worldwide, depressive disorders are the single largest contributor to the health burden among 10- to 24-year-olds.^1^ Most major episodes in young adulthood are preceded by earlier episodes during adolescence, although a substantial fraction of those experiencing depressive symptoms in adolescence appear to have recovered by early adulthood. The general understanding is that symptoms are rare in childhood but begin to rise around age 12 and peak in late adolescence (age 16–19), before decreasing in young adulthood.^2–4^ Research has recently focused on heterogeneity in trajectories of depressive symptoms, on the basis that classification of distinct subgroups may inform prognosis and development of interventions.^5–7^ Studies tend to identify a large group with low and stable symptoms throughout adolescence and smaller groups with moderate, high, increasing or decreasing trajectories. However, attempts to find a consensus on a taxonomy of depressive trajectories are complicated by marked differences in periods over which trajectories are estimated. A recent systematic review of trajectory-based studies of adolescent depression found only 3 (out of 20) studies that used a follow-up period encompassing the 12–20 age range, with the majority covering different subsets of the years prior to age 20 only.^5^ A trajectory group that appears homogeneous over a restricted developmental stage may in fact contain heterogenous groups whose trajectories diverge before or after the observation period. The median age at onset of major depressive episodes is the early-to-mid 20s^8^ and studies ending before this age cannot identify potentially distinct trajectories associated with differences that emerged during that period.^4^

## Predictors and outcomes of depression in young people

Several previous studies explored the risk factors associated with membership of different trajectory groups in depth, establishing that female gender is strongly associated with groups with higher symptoms, along with (less consistently) low socioeconomic status, stressful life events, substance use, conduct problems, lack of social/peer support, lack of a positive parental relationship, parental depressive symptoms^5,7^ and genetic risk.^9^ Much less attention has been paid to the later-life consequences of different trajectories,^3^ and most studies addressing this were focused on associations between trajectory and later psychiatric diagnoses.^7^ This study examines the consequences of adolescent depressive symptoms for education and employment-related outcomes in young adulthood. Specifically, for individuals aged 24, we examine whether they have a university degree and whether they are currently not in education, employment or training (NEET). The benefits of higher education qualifications to an individual, both financially and in terms of broader well-being, are well-documented, as are the social returns associated with lower public social welfare spending and higher tax revenue.^10^ Furthermore, young people who are NEET are regarded as a high-risk group for adverse health, personal and societal outcomes. Whereas degree attainment distinguishes around one-third of under-30s at the higher end of the socioeconomic spectrum,^10^ NEET status distinguishes just over one in ten 16- to 24-year-olds at the lower end of the socioeconomic spectrum.^11^ Understanding of the full burden of early mental health conditions requires knowledge of the impact not only on health but also on a range of social outcomes such as these, and can potentially have a major influence on economic calculations of the benefits of intervention programmes.^12^

A considerable body of research has explored the links between adolescent mental health conditions (including depression) and education and employment-related outcomes, but this literature typically considers mental health measures at just one or two time points. It is well established that adolescent emotional problems are negatively related to school grades/completion.^13,14^ The relationship with higher-education participation or completion is less clear with both significant^15^ and non-significant^16^ associations found. Adolescent depression has also been linked to poor young adult employment outcomes^16^ including NEET status.^14,17,18^ Mechanisms operating during school years discussed in this literature include impaired concentration, reduced motivation and disengagement;^13^ and truancy, long-term school absence, peer problems, disruptive classroom behaviour and substance use.^19^ Poor educational achievement and disengagement from school are themselves mediators for adult NEET status^17,19^ and continuation of mental disorders into young adulthood may also have current effects on ability to engage in employment or training, for example via reduced motivation or coping with stress during the job application process.^17,18^ However, it has also been argued that the associations with depression reflect confounding^14,16^ or bidirectionality, with evidence that academic problems^20^ and NEET status^18^ themselves have mental health consequences.

## Contribution of this study

Few studies (and none from the UK) have directly explored the association between trajectories of depressive symptoms in adolescence and subsequent education/employment outcomes, with only two following individuals from adolescence into young adulthood.^3,21^ It is clear that much remains to be learned about distinct trajectories of depressive symptoms that are (and are not) linked to poorer adult social outcomes and the timing at which different groups can be identified. The overarching aim of this study is to add to this small strand of literature and provide the first UK evidence on the educational and employment consequences associated with different trajectory profiles. Unlike most previous trajectory-based studies, we are able to estimate trajectories using nine measurements from pre-adolescence (age 11) to young adulthood (age 24) for a large population-based sample. These features of the data make it possible to identify heterogeneous trajectory groups that may be conflated in studies with shorter follow-up periods and/or smaller sample sizes. Previous work using the same data has analysed repeated measures of depressive symptoms^4,9^ or explored the relationship between one-off measures of depressive symptoms and educational outcomes/NEET status,^14^ but this is the first study using these data to test for the presence of heterogeneous trajectory groups over the full 13-year follow-up period and relate these trajectories to young adulthood outcomes. We also tested whether the associations between trajectory group and the distal outcomes are robust to controls for a set of baseline confounders suggested in the literature. Because of the fluctuating nature of depressive symptoms during adolescence, associations with educational/employment outcomes can be attenuated when symptoms are measured at only one time point. Using repeated measures may also aid early identification of at-risk individuals if distinct trajectories, as well as symptom severity, predict poor adult outcomes.^6^

Although scarcity of previous evidence hinders hypothesis formulation, insights from life-course epidemiological theory suggest three alternatives.^22^ First, it may be that the effects of depressive symptoms are time-limited, so that trajectories characterised by high current levels of symptoms at the time of NEET status measurement (age 24) and university study (ages 17–22) are the ones most strongly associated with the outcomes (recency hypothesis). Second, it may be that depressive symptoms at a particular developmental stage have consistent consequences for adult outcomes, regardless of the trajectory, because they coincide with a key transition period for education and/or employment (sensitive period hypothesis). Third, it may be that chronicity of symptoms matters, so that risks are greatest for those on trajectories with long spells of symptoms at or above a clinical level (accumulation of risk model).

## Method

### Participants

The Avon Longitudinal Study of Parents and Children (ALSPAC) was set up by enrolling pregnant women resident in Avon, UK with expected dates of delivery from 1 April 1991 to 31 December 1992. A total of 14 901 were alive at 1 year of age, with 14 691 constituting the full sample size available for this study.^23,24^ Ethical approval for the study was obtained from the ALSPAC Ethics and Law committee and the local research ethics committees. Individuals consented to participate in the study on the clear understanding that all measures would be used for research purposes only and not to inform decisions about their health (see supplementary File 1 available at https://doi.org/10.1192/bjo.2019.90 for further details).

### Measures

#### Exposure

##### Short Mood and Feelings Questionnaire (SMFQ)

The SMFQ^25^ is a 13-item self-reported questionnaire widely used for screening of depressive symptoms in adolescents. Individuals are asked to appraise each phrase as descriptive of their experiences ‘most of the time’, ‘sometimes’, or ‘not at all’ in the past 2 weeks. Total scores range between 0 and 26, with higher scores indicating more depressive symptoms. ALSPAC participants completed this questionnaire on nine occasions between the ages of approximately 11 and 24. Although there is no agreed cut-off, scores above 11 have been previously interpreted as indicating the presence of depression.^14^

#### Validators

##### Development And Well-Being Assessment (DAWBA)

The DAWBA^26^ is a semi-structured interview examining mental health symptoms and their impact on children aged 11–16 years. It can be used to assign psychiatric diagnoses following DSM-IV criteria. Diagnosis of depression was assessed at the 15-year visit.

##### Clinical Interview Schedule Revised (CIS-R)

The CIS-R^27^ is a structured instrument designed for use in general practice and community settings. Psychiatric diagnoses can be assigned following ICD-10 criteria. This includes depression, which was assessed at the 18-year visit.

##### Depression history

As part of the data collection at 23 years, participants were asked if they had ever been diagnosed with depression by a clinician.

##### Suicide attempts

Participants were asked whether they attempted suicide between 21 and 24 years.

#### Distal outcomes

University degree. We used self-reported information collected at the 23- and 24-year waves to create a binary variable indicating whether the participant obtained a university degree by age 24.

##### NEET

At age 24 participants were asked to complete a checklist of whether they were currently engaged in 11 different education/employment activities (see supplementary Table 1). Participants were classified as NEET if they indicated they were not engaged in any of the following: full- or part-time paid work; government training scheme; full-time education; self-employment; other self-reported activity indicating a job or training (for example maternity leave, part-time education). This definition is in line with the definition used by the UK Office for National Statistics.^11^

#### Potential confounders

##### Maternal post-natal depression

Mothers completed the Edinburgh Post-Natal Depression Scale^28^ 8 weeks after giving birth. This ten-item instrument is intended for pregnant women and new mothers. Total scores range between 0 and 30, with scores of 13 or above often interpreted as signifying depression.

##### Maternal education

At the child's 8-year clinic visit, mothers were asked whether they had finished compulsory school.

##### IQ

Child IQ was also measured at the 8-year visit using the Third Edition of the Weschler Intelligence Scale for Children.^29^ Verbal and non-verbal scores were averaged for the analyses.

##### Attitude towards school

Children were asked at age 11 whether they enjoyed school and whether they were afraid of school failure. Both variables were recoded into two categories (always/usually versus sometimes/never).

##### Educational attainment

We used school records to identify students performing at the expected level or above in English, maths, and science at age 11 (level 4 in key stage 2 national standardized tests).

### Statistical analyses

We conducted growth mixture modelling in Mplus v8,^30^ using the SMFQ measures as manifest variables with the timing of measurements fixed to the sample mean age at each measurement occasion (see supplementary Table 2). We considered sample-size-adjusted Bayesian Information Criterion (ssaBIC),^31^ Lo-Mendell-Rubin adjusted likelihood ratio test (LRT),^32^ bootstrap LRT test,^33^ size of smallest class and substantial interpretability to decide between models with different numbers of classes. We validated the identified trajectories by using three-step bias-adjusted growth mixture models^34^ to estimate their association with clinically diagnosed depression at age 15 and 18, history of depression by age 23, and suicide attempts between 21 and 24 years. This approach recognises that latent class categories can only be assigned probabilistically to participants. Further details of the statistical analysis are provided in supplementary File 1.

As shown below, there were large amounts of missing data in our analyses. Exclusion of individuals with missing data may result in unrepresentative samples, so we implement full-information-maximum-likelihood (FIML) estimation^35^ that utilises data from participants with partially, as well as fully, observed data. FIML produces unbiased estimates under the assumption that data are missing at random (MAR). Since MAR is unlikely to hold perfectly in this application, we assessed the sensitivity of our results to the exclusion of participants with several patterns of missing data. Our main first-stage latent class model utilised the maximum possible sample of respondents compatible with FIML estimation: those with at least one SMFQ measurement (*n* = 9399). We also performed analyses separately for boys and girls; restricting the sample to participants with either three or more, or all nine SMFQ measures; and excluding the cases of 183 individuals with unusual reporting patterns. In the second-stage models that use latent class membership to predict the distal outcomes, we compared results for participants where outcomes were observed and those where FIML was used to incorporate participants with missing outcome data. Mplus does not allow for FIML modelling of missing covariate data, hence the sample size for the adjusted models was reduced from *n* = 9399 to *n* = 4501.

## Results

The earliest SMFQ measure was taken at an average age of 10.6 years and was reported by 7364 children, whereas the most recent measure was obtained at an average age of 23.8 years and was completed by 3915 participants. A wide range of response patterns were observed, (see supplementary Tables 2–4). Correlations between SMFQ measures showed stronger associations for measurements taken at similar ages (see supplementary Table 5). Descriptive characteristics for a range of subsamples with different patterns of missing data are presented in Table 1. The first column provides statistics for all data available on the full ALSPAC sample (*n* = 14 691). Many variables are only partially observed for this sample, but it is possible to compare the full cohort with the analysis sample of those with at least one SMFQ measure (second column; *n* = 9399). The analysis sample is similar in terms of maternal age, has a higher percentage of female participants, maternal educational attainment and children's academic attainment at age 11, and a lower rate of postnatal depression.

**Table 1:** Descriptive characteristics of study participants^a^

	Full ALSPAC sample (*n* = 14691)	One or more SMFQ measures (*n* = 9399)	Three or more SMFQ measures (*n* = 7084)	Nine SMFQ measures (*n* = 1031)	One or more SMFQ measures + full covariate data (*n* = 4501)
Female participants					
%	48.70	52.20	54.80	67.00	51.10
Total *n*	14684	9394	7081	1031	4501
Maternal age					
Mean (s.d.)	28.4 (4.82)	28.9 (4.59)	29.3 (4.49)	30.4 (4.33)	29.4 (4.40)
Total *n*	11809	8269	6387	948	4501
Maternal postnatal depression					
%	10.10	9.09	8.44	7.59	7.84
Total *n*	11691	8199	6342	948	4501
Mother finished compulsory school					
%	49.10	60.30	67.50	82.70	74.60
Total *n*	12456	9162	6965	1013	4501
Child's IQ					
Mean (s.d.)	103 (15.5)	103 (15.3)	104 (15.1)	110 (13.9)	104 (15.1)
Total *n*	7402	6916	5859	988	4501
Child enjoys at school					
%	83.20	83.90	84.90	87.50	84.30
Total *n*	7362	6876	5846	974	4501
Child afraid of school failure					
%	5.80	5.68	5.74	5.56	5.51
Total *n*	7288	6811	5797	972	4501
Child achieved school goals at age 11					
%	70.80	78.00	81.50	90.90	83.00
Total *n*	12164	8101	6159	908	4501
Clinical diagnosis at 15					
%	1.66	1.66	1.59	1.21	1.57
Total *n*	5368	5304	5030	995	3303
Clinical diagnosis at 18					
%	7.89	7.82	7.86	8.09	7.85
Total *n*	4563	4525	4248	976	2715
History of depression by 23					
%	15.10	15.20	14.60	13.50	13.80
Total *n*	3955	3934	3743	1028	2208
Suicide attempts between age 21 and 24					
%	4.41	4.41	4.23	2.04	3.99
Total *n*	3924	3924	3733	1028	2203
Child had university degree by age 24					
%	56.20	56.20	56.90	69.30	59.00
Total *n*	4208	4207	3975	1030	2360
Child was NEET at age 24					
%	9.13	9.09	8.67	7.01	7.09
Total *n*	3954	3951	3736	1027	2213

ALSPAC, Avon Longitudinal Study of Parents and Children; SMFQ, Short Mood and Feelings Questionnaire; NEET, not in education, employment or training.

a. Total *n* for each variable is included as there are missing data.

Models that adjust for potential confounding from these covariates allow us to assess the sensitivity of results to these dimensions of sample composition. Our main analyses are conducted on the sample in the second column and the first-stage latent class model uses FIML to include participants with missing SMFQ scores. Supplementary Table 6 show the proportions of missing data for each trajectory group. Even if MAR is unlikely to hold perfectly, the descriptive statistics in the third and fourth columns of Table 1 suggest a FIML approach is preferable to a complete-case analysis. These statistics show that restricting the sample used to conduct the latent class analysis results in an increasingly unrepresentative sample when three or more SMFQ measures are required (*n* = 7084), and particularly when all nine measurements are required (*n* = 1031).

Statistics in the first two columns reveal a large amount of missing data on our NEET and degree outcome measures, which were measured at the end of the observation period. Among those with valid outcome data, 56.2% had a degree and 9.1% were NEET at age 24. These proportions indicate noticeably higher levels of advantage than 2016 national estimates, which put the per cent of the UK population under 30 with degrees at 34% and the per cent of 24-year-olds in England with NEET status at 14.0%.^10,11^ The final column in Table 1 provides statistics for the sample used to construct adjusted estimates (*n* = 4051). Its composition is generally similar to the main analysis sample in the second column although proportions of maternal education, child academic attainment at 11 and degree attainment are higher and NEET status is less prevalent. The consequences of this final sample selection at the second stage can be assessed by comparing unadjusted results with those from the full-analysis sample.

### Choice of number of classes

We fitted models using a quadratic polynomial for shapes and adding classes incrementally. The variance of the intercept term was fixed to zero because for models with more than two classes, the classes explained the variation in the intercept. Solutions with seven or more classes yielded very small class sizes and therefore we did not consider those further. Adding classes to the model generally resulted in a better model fit (smaller ssaBIC) and was supported by both tests up to five classes (see supplementary Table 7). The entropy of this model (0.736) suggests some degree of uncertainty in the classification.

Adding a sixth class still improved model fit, however, the Lo-Mendell-Rubin test suggested that the six-class solution did not significantly improve fit over the five-class solution. Furthermore, although all trajectories in the five-class solution had a meaningful interpretation, this was less clear for the six-class solution, which split the category with the highest symptoms from the five-class model into two categories with different severity levels, one of them accounting for only 2.1% of the sample. Therefore, the remaining analyses of this paper focus on the five-class solution.

**Fig. 1:**
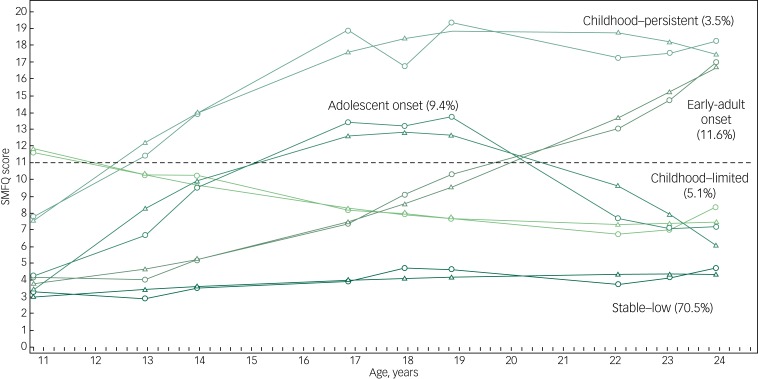
Five-class trajectory solution.

Figure 1 displays the observed (in circles) and estimated (triangles) trajectories for each class. The largest group found had a stable trajectory of low depressive symptoms (we use the label ‘stable–low’ for this category, which includes 70.5% of the sample). The second largest group (‘early-adult onset’, 11.6%) was characterised by an increasing trend in depressive symptoms, reaching an average depression score above 11 (the conventional SMFQ threshold) at age 22 and exceeding 16 by age 24. The third largest group, labelled as ‘adolescent onset’ (9.4%), began with low scores and, reached full-threshold scores by age 17 before declining sharply after age 19. The fourth group (‘childhood–limited’, 5.1%) showed the highest average depression score at onset, which consistently decreased over time. Finally, the smallest trajectory (‘childhood–persistent’, 3.5%) yielded the highest average depression scores from age 13 and showing increasing symptoms until age 19.

Short Mood and Feelings Questionnaire (SMFQ) scores above 11 are often interpreted as signifying depression. Circles represent observed means, while triangles represent predicted means at each occasion.

Table 2 displays the proportion of depression at age 15 and 18 years, depression history by age 23, and suicide attempts between ages 21 and 24 for each trajectory. The rankings in terms of the risks changed over time in accordance with the shape of the trajectory profiles, with childhood–persistent yielding the highest risks on all validators at all ages and early-adult onset showing the second largest risks only for the adult validators. These results suggest that the trajectories identified are clinically meaningful and that they could not be distinguished on the basis of a diagnostic measure taken at a single point in time. The five trajectory groups also differed significantly in their composition in terms of covariates (supplementary Table 8).

**Table 2:** Validation of the five-class solution (*n* = 9399)^a^

Class	Proportion (95% CI)			
	Age 15 (DAWBA)	Age 18 (CIS-R)	Age 23 (depression history)	Suicide attempts (age 21–24)
Stable–low	0.004 (0–0.008)	0.011 (0.003–0.019)	0.028 (0.024–0.032)	0
Childhood–limited	0.053 (0.018–0.088)	0.070 (0.013–0.127)	0.172 (0.084–0.260)	0.052 (0–0.107)
Adolescent onset	0.053 (0.024–0.082)	0.270 (0.209–0.331)	0.284 (0.213–0.355)	0.024 (0–0.059)
Early-adult onset	0.026 (0.006–0.046)	0.159 (0.112–0.206)	0.450 (0.391–0.509)	0.164 (0.127–0.201)
Childhood–persistent	0.081 (0.024–0.138)	0.557 (0.439–0.675)	0.627 (0.507–0.747)	0.401 (0.289–0.513)
Total (observed)	0.017	0.078	0.151	0.044

DAWBA, Development And Well-Being Assessment; CIS-R, Clinical Interview Schedule Revised.

a. Numbers in the table are proportions (95% CIs) estimated via a three-step bias corrected logistic regression procedure with trajectory group as the predictor and the validator as the dependent variable. Missing data is handled by FIML.

Sensitivity analyses restricted to boys, girls, participants with at least three SMFQ measures, participants with all nine SMFQ measures, or excluding unusual reporting patterns further supported the five-class solution, although the distribution of participants across classes showed some variations (supplementary Figs 1–5).

### Associations with distal outcomes

Table 3 shows the results of the three-step bias-adjusted models with the trajectories as the exposure and two distal outcomes at age 24. The first two columns for each outcome compare unadjusted results for samples including and excluding participants with missing outcome data. Participants in the childhood–limited group were more likely than those in the stable–low group to be NEET at 24 but do not differ in terms of degree attainment. Conversely, those in the adolescent onset group were less likely to have attained a degree but no different to the stable–low group in NEET risk. The remaining two groups – early-adult onset and childhood–persistent – had increased risks of poor outcomes on both measures. Comparing effect magnitude, NEET risks were consistently greatest for the childhood–persistent group. Rates of degree attainment among the adolescent onset group, although lower than the stable–low group, were consistently higher than the early-adult onset and childhood– persistent groups.

**Table 3:** Results of growth mixture models with a distal outcome^a^

Class	University degree by age 24				NEET status by age 24			
	Full sample		Sample with valid covariate data		Full sample		Sample with valid covariate data	
	All (unadjusted)	Observed DV only (unadjusted)	Unadjusted	Adjusted^b^	All (unadjusted)	Observed DV only (unadjusted)	Unadjusted	Adjusted^b^
Childhood–limited, OR (95% CI)	0.77 (0.49–1.20)	0.75 (0.46–1.21)	0.87 (0.48–1.56)	1.30 (0.57–2.97)	3.49 (1.87–6.51)	4.24 (2.12–8.47)	3.64 (1.47–9.02)	3.15 (1.27–7.81)
Adolescent onset, OR (95% CI)	0.54 (0.41–0.71)	0.68 (0.49–0.94)	0.73 (0.48–1.13)	0.68 (0.43–1.06)	1.43 (0.69–2.94)	1.64 (0.75–3.60)	1.69 (0.64–4.46)	1.73 (0.68–4.45)
Early-adult onset, OR (95% CI)	0.23 (0.13–0.40)	0.53 (0.41–0.69)	0.55 (0.38–0.80)	0.54 (0.36–0.80)	3.55 (2.35–5.35)	4.03 (2.48–6.53)	4.06 (2.16–7.65)	3.70 (1.94–7.04)
Childhood–persistent, OR (95% CI)	0.71 (0.51–0.98)	0.20 (0.11–0.38)	0.06 (0.01–0.38)	0.08 (0.02–0.37)	6.12 (3.57–10.5)	7.50 (4.17–13.5)	6.16 (2.51–15.1)	5.17 (1.95–13.7)
*N*	9399	4207	4501	4501	9399	3951	4501	4501

NEET: not in education, employment or training; DV, dependent variable.

a. The full sample is the sample with one or more SMFQ measures. The reference category is stable–low.

b. Adjusted for gender, IQ (continuous), maternal postnatal depression, maternal education, attitude towards school and academic results at age 11 as potential confounders.

The third column for each outcome can be compared with the first column to isolate the impact of dropping participants with any missing data on covariates. In most cases the results are very similar, the evidence of a lower proportion of degree attainment in the adolescent onset compared with the stable–low group (odds ratio (OR)=0.54, 95% CI 0.41–0.71) becomes unclear with the restricted sample (OR=0.73, 95% CI 0.48–1.13). Comparison of the third and fourth columns for each outcome provides evidence on the extent to which the unadjusted ORs reflect confounding from baseline characteristics. Estimates changed only marginally, suggesting that differences in the outcomes across groups are not driven by pre-existing differences in maternal characteristics, cognitive ability or educational experiences by age 11.

## Discussion

### Trajectories of depressive symptoms

In this study, we identified five groups of individuals with different trajectories of depressive symptoms between late childhood (approximately age 11) and early adulthood (approximately age 24) and explored the associations of each group with educational and employment outcomes. Four of these trajectories have been widely reported in previous studies,^5–7^ low (here stable–low); high (childhood–persistent); increasing (early-adult onset); and decreasing (childhood–limited). Conversely, the adolescent onset group with symptoms that rise to a peak around age 17–19 before declining again is more unusual, although it has been found in at least one trajectory-based study^2^ and is consistent with studies examining aggregate patterns for the whole population.^4^ Notably, symptoms in the adolescent onset, childhood–persistent and early-adult onset groups all follow consistently increasing trajectories, albeit at different levels, between 11 and 19 years.

Most previous studies did not survey individuals into their 20s and, as a result, may conflate the adolescent onset group with one of the other increasing-trajectory groups. One contribution of this study, therefore, is to provide evidence of heterogeneity among those whose symptoms rise through to late adolescence and identify a sizable group (9.4% of the sample) whose symptoms remit in early adulthood, in contrast to other groups whose symptoms persist or worsen. The increasing symptoms trajectories found in many previous studies of adolescent depression may in fact consist of two different subgroups with quite distinct prognoses. Whether these differences are the result of different experiences in late adolescence (for example receipt of mental health treatment) or different pre-existing characteristics is an intriguing question for future research.

### Social outcomes

We then went on to examine the association of trajectory groups with two young adult social outcomes: attainment of a university degree and NEET status at 24 years. All four groups with a full-threshold period of depressive symptoms were at greater risk of at least one poor adult outcome compared with the stable–low group. This finding is in line with a previous study that found three trajectories between ages 16 and 30 and showed that those in the ‘moderate–decreasing’ and ‘high–stable’ groups were at progressively greater risk of poor educational attainment and low income at age 30 than individuals in a ‘low–decreasing’ group.^21^ These results suggest that studies examining associations between depressive symptoms measured on a single occasion in adolescence and adult outcomes will tend to understate the later risks associated with depressive symptoms in young people. Such studies are also unable to examine hypotheses regarding the importance of symptom timing and chronicity.

### Interpretation of our findings

We highlighted three hypotheses derived from life-course epidemiology (recency, accumulation and sensitive periods) and found some support for each one. The distinction is somewhat blurred for the degree outcome, which is the culmination of a lengthy process that took place between 17 and 22 years for most participants. The recency hypothesis states that the outcome will be predicted most strongly by symptoms experienced just before or at the time of its measurement. This is supported by the fact that the three groups with full-threshold symptoms at some point between ages 17 and 22 all had lower odds of attaining a university degree than the stable–low group. Further, two of these groups – childhood–persistent and early-adult onset – had continued full-threshold levels of symptoms at ages 22–24 and higher odds of being NEET at age 24. Furthermore, consistently with the sensitive period hypothesis, all children with full-threshold levels of symptoms between 11 and 13 years were at higher risk of NEET status at 24, regardless of whether those symptoms tended to worsen over time (childhood–persistent) or improve (childhood–limited). Finally, the group with sustained clinical symptom levels throughout the longest period of time (childhood–persistent) showed the poorest outcomes in early adulthood, which supports the accumulation hypothesis.

Our results suggest that full-threshold levels of symptoms at any age between 11 and 24 are a marker for poor adult outcomes in at least one domain. We have argued that, perhaps unsurprisingly, measurement of adolescent depressive symptoms on any single occasion will tend to yield false negatives. A valuable insight from our trajectory-based analysis, however, is that identification could be improved by drawing on information on whether an individual's mood has worsened over the previous 1–3 years, in addition to information on the level of current symptoms.

For instance, the early–adult onset group only exhibits full-threshold levels of symptoms after age 20 but can be distinguished from the stable–low group as early as 17. Moreover, increasing symptoms between 11 and 13 years characterise the adolescent onset and childhood–persistent groups, but the former only reaches full-threshold levels of symptoms until age 17. Hence an implication of our findings is that if practitioners were to collect and interpret retrospective information on symptom trajectories, as well as symptom levels, in early-to-mid adolescence more individuals could potentially be effectively targeted for preventative interventions. Many preventative interventions for internalising disorders in young people have been trialled, mostly delivered within the school setting and psychologically centred. The evidence suggests that, on average, these interventions are effective but with considerable heterogeneity in effects and larger effects for targeted interventions.^36^

A number of studies have argued that bivariate relationships between depressive symptoms in young people and educational and employment outcomes reflect confounding by common factors such as childhood socioeconomic conditions and academic aptitude.^14–16^ The stability of our results when covariates are added to the models provides little support for this explanation perhaps because, as explained above, trajectory group differences are likely to be larger and more precisely estimated than single time point effects. However, there is also evidence that bivariate associations may be driven by co-occurring disorders such as anxiety, substance use, delinquency and psychotic experiences, although interpretations vary as to whether these are considered confounders^14,16,18^ or mediators.^19^ We did not examine this type of explanation in this study but we note that preventative mental health interventions often have a positive impact on a range of disorders^36^ and so may be effective even if depressive symptoms are not causal in a narrow sense. Regardless of the mechanism targeted by interventions, our finding of problematic trajectories characterised by symptoms that remain high or increase into early adulthood adds further support to calls for the elimination of discontinuities in provision of mental health services at age 18.^37^

### Strengths and limitations

This study is rare in the literature in drawing on a large population-based sample with high-frequency repeated measures of depressive symptoms spanning early adolescence to early adulthood and measures of key young adult outcomes of social importance. However, it has several limitations. First, there are large amounts of missing data on many of the analysis variables. We dealt with this via FIML methods and sensitivity analyses to explore the robustness of the results. However, we acknowledge that the necessary MAR assumption is unlikely to hold perfectly and there may be remaining biases in the results.

Second, this is an observational study and therefore the associations that we found are prone to confounding. Adjustment for a set of baseline covariates commonly discussed in the literature had little impact on the results, but they do not encompass all potential sources of unobserved heterogeneity. Third, this study did not explore the role of any time-varying covariates. Future research should explore factors driving the trajectory shapes, including co-occurring mental disorders, treatment (or lack of it) for mental health disorders and a range of social processes.

Fourth, some research has found evidence of heterogeneity within the NEET group and argued that parents/homemakers should be grouped with those engaged in work or training.^17^ Here we simply use the UK government definition of NEET and note that parents/homemakers make up only a small fraction of our NEET group. Fifth, our findings are limited to our sample and might not generalise to other nations, ethnicities or cultures.

## Data Availability

J.A.L.-L., R.M.P., E.W., K.T., G.H. and A.S.F.K. had full access to the study data.

## Supplementary material

For supplementary material accompanying this paper visit http://dx.doi.org/10.1192/bjo.2019.90. click here to view supplementary material
