# Supplementary material for: Trajectories of depressive symptoms and adult educational and employment outcomes
Source: BJPsych Open. 2019 Dec 12;6(1):e6. doi: 10.1192/bjo.2019.90 (PMC7001468; doi:10.1192/bjo.2019.90)

# Supplementary materials

## Supplementary File 1 Methods

## Participants

The initial number of pregnancies enrolled is 14,541. Of these initial pregnancies, there was a total of 14,676 foetuses, resulting in 14,062 live births and 13,988 children who were alive at 1 year of age. When the oldest children were approximately 7 years of age, an attempt was made to bolster the initial sample with eligible cases who had failed to join the study originally. As a result, when considering variables collected from the age of seven onwards (and potentially abstracted from obstetric notes) there are data available for more than the 14,541 pregnancies mentioned above. The number of new pregnancies not in the initial sample (known as Phase I enrolment) that are currently represented on the built files and reflecting enrolment status at the age of 24 is 913 (456, 262 and 195 recruited during Phases II, III and IV respectively), resulting in an additional 913 children being enrolled. The total sample size for analyses using any data collected after the age of seven is therefore 15,454 pregnancies, resulting in 15,589 foetuses. Of these 14,901 were alive at 1 year of age. ALSPAC parents and children have been followed longitudinally, with mothers, mothers’ partners, and children providing data through postal questionnaires and clinic visits. The study website contains full details of all available data (<http://www.bristol.ac.uk/alspac/researchers/our-data/>).

## Statistical analyses

As a first step of the growth mixture modelling, we fitted a latent class model on the SMFQ measures alone. After examining different trajectory shapes, we chose a quadratic polynomial including an intercept (I), slope (S), and quadratic (Q) growth factor for each class based on conceptual (non-linear mood patterns are common throughout adolescence) and statistical reasons. We estimated the mean of each growth factor separately for each class but constrained the growth factor variances to be equal across classes. Residual variances were assumed to be longitudinally invariant in the main analyses (a more parsimonious model than considering different variance parameters at each time point, which led to the same class solution). After determining the optimal number of classes, the second stage involved fitting logistic regression models to incorporate university graduation or NEET as distal outcomes, again using a bias-adjusted three-step approach, both with and without adjusting for sex, IQ, maternal post-natal depression, maternal education, attitude towards school, and educational achievement at age 11 as potential confounders.

## Supplementary Table 1. NEET classification

|  | Number of NEET in activity | % of all NEETs in activity |
| --- | --- | --- |
| Any activity (total) | 359 | 100.0% |
| In irregular or occasional work | 62 | 17.3% |
| Unemployed and looking for work | 175 | 48.7% |
| Unable to work through sickness/disability | 69 | 19.2% |
| Doing voluntary work | 43 | 12.0% |
| A full/part-time carer | 18 | 5.0% |
| Other: Stay-at-home parent /Homemaker | 27 | 7.5% |
| Other: Currently travelling /Waiting to start new job/education | 19 | 5.3% |
| Other: Not specified | 36 | 10.0% |

Notes: Individuals were presented with a yes/no checklist of activities and asked “Are you currently..? (Please cross one box on each line)”. Individuals were classified as “not NEET” if they answered “yes” to any of: In full-time paid work (30+ hours per week); In part-time paid work (<30 hours per week); Doing a modern apprenticeship or other government supported training/work-experience scheme; In full-time education; or Self-employed. In addition, individuals were classified as “not NEET” if they answered “yes” to Other activity and went on to specify a free-text response given one of the following codes by the survey administrators: Part-time education; Doing a Phd; Teacher training; Student nurse; Placement student; Maternity leave; Internship; Employed but looking for work. Individuals were classified as “NEET” if they had not indicated any of the employment/training activities listed above but had completed at least one checkbox.

The table gives the number of cases classified as NEET recording each possible activity. Responses were not mutually exclusive, so percentages sum to more than 100%. For example, although 48.7% indicated Unemployed, seeking work and 19.2% indicated Unable to work through sickness/disability, the combined total with either of these responses was 65.7% (N = 236). Results are presented for those with at least one SMFQ score (a subset of the N=9399 analysis sample) because only 3 cases recorded a valid NEET status but had no valid SMFQ scores.

## Supplementary Table 2. Descriptive statistics of SMFQ measures at each time point

| Mean age (SD) | Age range | Sample size | Median | Mean score | SD of scores | α | Source |
| --- | --- | --- | --- | --- | --- | --- | --- |
| 10.6 (0.26) | 9.83-12.2 | 7,364 | 3 | 4.04 | 3.51 | .797 | Clinic |
| 12.8 (0.22) | 11.3-14.2 | 6,716 | 3 | 3.97 | 3.86 | .842 | Clinic |
| 13.8 (0.19) | 12.6-15.1 | 6,019 | 4 | 4.92 | 4.49 | .865 | Clinic |
| 16.7 (0.24) | 16.4-18.1 | 4,997 | 4 | 5.91 | 5.64 | .908 | Questionnaire |
| 17.8 (0.35) | 16.3-19.6 | 4,497 | 5 | 6.58 | 5.25 | .897 | Clinic |
| 18.7 (0.48) | 17.9-20.0 | 3,334 | 5 | 6.82 | 5.93 | .906 | Questionnaire |
| 21.9 (0.51) | 20.9-23.2 | 3,305 | 4 | 5.70 | 5.58 | .915 | Questionnaire |
| 22.9 (0.50) | 21.9-24.2 | 3,856 | 5 | 6.21 | 5.55 | .906 | Questionnaire |
| 23.8 (0.51) | 22.8.25.1 | 3,915 | 5 | 7.03 | 6.06 | .913 | Questionnaire |

α: Coefficient alpha to estimate reliability of test scores

## Supplementary Table 3. Timing of first valid SMFQ observation

| Age at first valid SMFQ | Freq. | Percent | Cum. Percent |
| --- | --- | --- | --- |
| 10.6 | 7,364 | 78.35 | 78.35 |
| 12.8 | 669 | 7.12 | 85.47 |
| 13.8 | 143 | 1.52 | 86.99 |
| 16.7 | 616 | 6.55 | 93.54 |
| 17.8 | 277 | 2.95 | 96.49 |
| 18.7 | 82 | 0.87 | 97.36 |
| 21.9 | 73 | 0.78 | 98.14 |
| 22.9 | 110 | 1.17 | 99.31 |
| 23.8 | 65 | 0.69 | 100 |
| Total | 9,399 | 100 |  |

## Supplementary Table 4. Missing data patterns for SMFQ measurements

| Pattern | Observed SMFQ scores, t = 1,…,9 | N | Percent | Cum. Percent |
| --- | --- | --- | --- | --- |
| 1 | All waves t=1 to t=9 | 1031 | 11.0 | 11.0 |
| 1 – 0 | Consecutive waves from t=1 to t<9 | 2563 | 27.3 | 38.2 |
| 0 – 1 | Consecutive waves from t>1 to t=9 | 285 | 3.0 | 41.3 |
| 0 – 1 – 0 | Consecutive waves from t>1 to t<9 | 912 | 9.7 | 51.0 |
| 1 – 0 – 1 | Intermittent response from t=1 | 3770 | 40.1 | 91.1 |
| 0 – 1 – 0 – 1 | Intermittent response from t>1 | 838 | 8.9 | 100 |
| Total |  | 9,399 | 100 |  |

## Supplementary Table 5. Pair-wise correlations between SMFQ measurements at different time points

|  | dep11 | dep13 | dep14 | dep17 | dep18 | dep19 | dep22 | dep23 | dep24 |
| --- | --- | --- | --- | --- | --- | --- | --- | --- | --- |
| dep11 | 1 |  |  |  |  |  |  |  |  |
| dep13 | 0.449 | 1 |  |  |  |  |  |  |  |
| dep14 | 0.406 | 0.624 | 1 |  |  |  |  |  |  |
| dep17 | 0.075 | 0.184 | 0.248 | 1 |  |  |  |  |  |
| dep18 | 0.143 | 0.244 | 0.311 | 0.347 | 1 |  |  |  |  |
| dep19 | 0.081 | 0.158 | 0.192 | 0.456 | 0.340 | 1 |  |  |  |
| dep22 | 0.068 | 0.143 | 0.199 | 0.406 | 0.323 | 0.472 | 1 |  |  |
| dep23 | 0.037 | 0.116 | 0.169 | 0.372 | 0.313 | 0.439 | 0.546 | 1 |  |
| dep24 | 0.036 | 0.114 | 0.172 | 0.345 | 0.305 | 0.419 | 0.500 | 0.605 | 1 |

## Supplementary Table 6. Estimated percentages of missing data, by trajectory group

| Variable | Stable low (70.5%) | Childhood limited (5.1%) | Adolescent onset (9.4%) | Early adult onset (11.6%) | Childhood persistent (3.5%) | All  (100%) |
| --- | --- | --- | --- | --- | --- | --- |
| SMFQ age 11 | 21.5% | 6.38% | 25.2% | 24.4% | 32.2% | 21.7% |
| SMFQ age 13 | 29.1% | 21.9% | 26.0% | 28.0% | 31.9% | 28.5% |
| SMFQ age 14 | 37.0% | 26.3% | 30.0% | 34.0% | 42.9% | 36.0% |
| SMFQ age 17 | 48.8% | 49.7% | 35.0% | 37.4% | 44.7% | 46.8% |
| SMFQ age 18 | 54.4% | 48.7% | 40.2% | 41.0% | 53.8% | 52.2% |
| SMFQ age 19 | 66.9% | 64.5% | 54.1% | 51.1% | 63.0% | 64.5% |
| SMFQ age 22 | 67.2% | 63.0% | 57.5% | 47.4% | 67.4% | 64.8% |
| SMFQ age 23 | 61.7% | 63.0% | 52.4% | 34.4% | 59.3% | 59.0% |
| SMFQ age 24 | 61.2% | 63.0% | 54.5% | 29.7% | 57.1% | 58.3% |
| Degree by 24 | 58.1% | 59.7% | 48.4% | 29.2% | 56.0% | 55.2% |
| NEET at 24 | 60.9% | 61.7% | 53.9% | 29.1% | 56.8% | 58.0% |
| Any covariate | 52.1% | 52.3% | 49.3% | 52.3% | 59.0% | 52.1% |

Note. Trajectory group membership is not observed deterministically but is estimated probabilistically from the first-stage latent class model. Numbers in the table are proportions estimated from a three-step bias-adjusted auxiliary logistic regression model with trajectory groups as the predictors and binary indicators for whether the value of the specified variable(s) is missing as the dependent variable. N = 9,399 in all models. Any covariate indicates at least one value is missing from gender, maternal education, maternal postnatal depression, IQ, whether the child enjoyed school at 11, was afraid of failure at 11 and reached the expected academic level at 11.

## Supplementary Table 7. Growth mixture modelling results using different class solutions

| k | NP | ssaBIC | Smallest class | Entropy | LMR(p) | BLRT(p) |
| --- | --- | --- | --- | --- | --- | --- |
| 1 | 10 | 253634 | 1 | - | - | - |
| 2 | 11 | 252306 | 0.13 | 0.79 | <.001 | <.001 |
| 3 | 15 | 251346 | 0.08 | 0.74 | <.001 | <.001 |
| 4 | 19 | 250909 | 0.05 | 0.75 | .104 | <.001 |
| **5** | **23** | **250484** | **0.03** | **0.74** | **.001** | **<.001** |
| 6 | 27 | 250230 | 0.02 | 0.75 | .097 | <.001 |

k: number of classes; NP: number of parameters; ssaBIC: sample size adjusted Bayesian Information Criterion; LMR: Lo-Mendell-Rubin likelihood ratio test; BLRT: bootstrap likelihood ratio test

## Supplementary Table 8. Proportions (and 95% CIs) for key variables in each of the five trajectories

|  | **Stable Low** | **Childhood  Limited** | **Adolescent  Onset** | **Early Adult  Onset** | **Childhood  Persistent** |
| --- | --- | --- | --- | --- | --- |
| *Variables related to the mother* | | | | | |
| Post-natal depression | .075  (.067 to .083) | .116  (.069 to .163) | .095  (.060 to .130) | .141  (.106 to .176) | .192  (.129 to .255) |
| Finished compulsory school | .580  (.566 to .594) | .561  (.494 to .628) | .666  (.613 to .719) | .629  (.582 to .676) | .419  (.341 to .497) |
| *Child core variables* | | | | | |
| Females | .448  (.434 to .462) | .509  (.440 to .578) | .785  (.736 to .834) | .669  (.622 to .716) | .833  (.770 to .896) |
| IQ>100 | .702 (.688 to .716) | .518  (.449 to .587) | .705 (.656 to .754) | .725  (.680 to .770) | .682  (.609 to .755) |
| *Education at age 11* | | | | | |
| Enjoy school | .857  (.845 to .869) | .714  (.643 to .785) | .836  (.789 to .883) | .814  (.771 to .857) | .730  (.648 to .812) |
| Afraid of school failure | .045  (.037 to .053) | .133  (.082 to .184) | .078  (.045 to .111) | .068  (.039 to .097) | .083  (.030 to .136) |
| Performed at expected level | .791  (.779 to .803) | .548  (.475 to .621) | .834  (.787 to .881) | .819  (.778 to .860) | .627  (.549 to .705) |
| *Distal outcomes by age 24* | | | | | |
| University degree | .608  (.586 to .630) | .543  (.484 to .602) | .524  (.450 to .598) | .457  (.398 to .516) | .261  (.151 to .371) |
| NEET status | .057  (.045 to .069) | .174  (.094 to .254) | .079  (.030 to .128) | .176  (.133 to .219) | .270  (.170 to .370) |

NEET: Not in Employment, Education or Training; IQ: Intelligence Quotient.

## Supplementary Figure 1. Five-class solution for boys (N=4495)


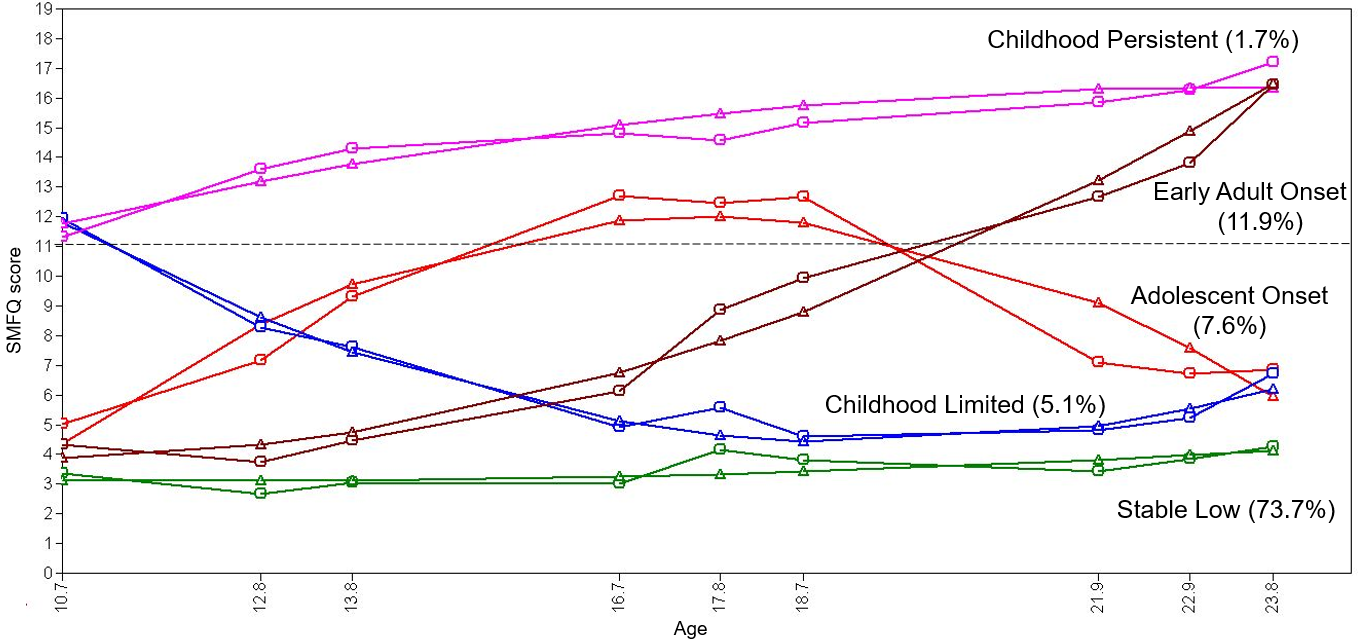


## Supplementary Fig 2. Five-class solution for girls (N=4899)


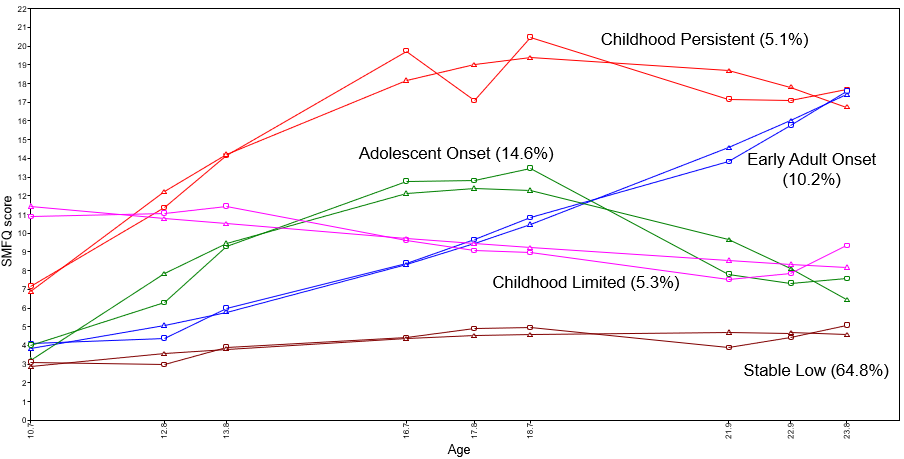


## Supplementary Fig 3. Five-class solution for participants with 3 or more SMFQ measures (N=7084)


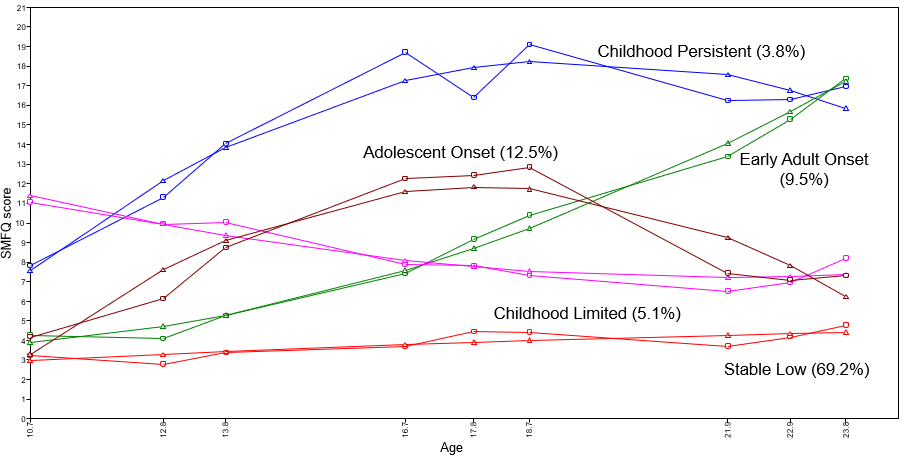


## Supplementary Fig 4. Five-class solution for participants with all 9 SMFQ measures (N=1031)


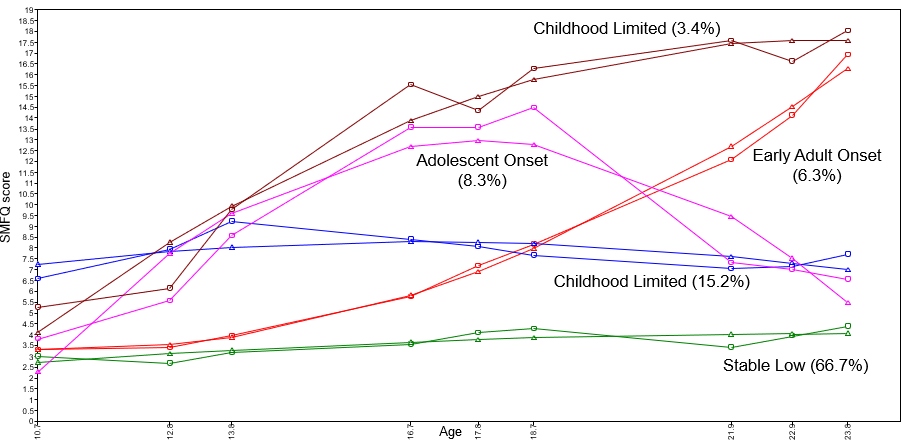


## Supplementary Fig 5. Five-class solution excluding unusual response patterns at age 18 (N=9398)

We spotted a spike in the distribution of SMFQ total scores at age 18, which was caused by a group of 183 participants responding ‘Sometimes true’ to every question in this and other related questionnaires. To test the robustness of our results, we ran further sensitivity analyses setting those participants as non-responders for that time point.


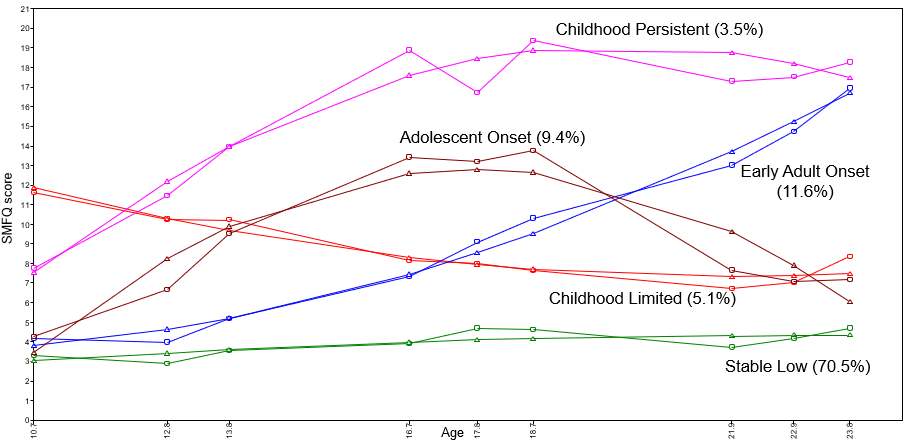

Supplement: Supplementary file 1 [file S2056472419000905sup001.docx]
